# 3D-Printed High-Pressure-Resistant Immobilized Enzyme Microreactor (μIMER) for Protein Analysis

**DOI:** 10.1021/acs.analchem.1c05232

**Published:** 2022-06-09

**Authors:** Tobias Rainer, Anna-Sophia Egger, Ricarda Zeindl, Martin Tollinger, Marcel Kwiatkowski, Thomas Müller

**Affiliations:** † Institute of Organic Chemistry and Center for Molecular Biosciences (CMBI), Leopold-Franzens University Innsbruck, 6020 Innsbruck, Austria; ‡ Institute of Biochemistry and Center for Molecular Biosciences (CMBI), Leopold-Franzens University Innsbruck, 6020 Innsbruck, Austria

## Abstract

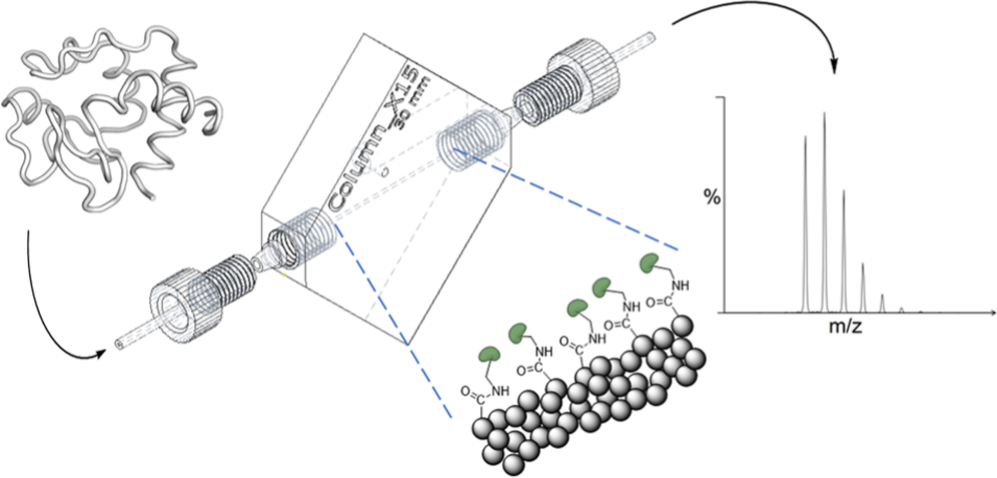

Additive manufacturing (3D printing) has greatly revolutionized the way researchers approach certain technical challenges. Despite its outstanding print quality and resolution, stereolithography (SLA) printing is cost-effective and relatively accessible. However, applications involving mass spectrometry (MS) are few due to residual oligomers and additives leaching from SLA-printed devices that interfere with MS analyses. We identified the crosslinking agent urethane dimethacrylate as the main contaminant derived from SLA prints. A stringent washing and post-curing protocol mitigated sample contamination and rendered SLA prints suitable for MS hyphenation. Thereafter, SLA printing was used to produce 360 μm I.D. microcolumn chips with excellent structural properties. By packing the column with polystyrene microspheres and covalently immobilizing pepsin, an exceptionally effective microscale immobilized enzyme reactor (μIMER) was created. Implemented in an online liquid chromatography-MS/MS setup, the protease microcolumn enabled reproducible protein digestion and peptide mapping with 100% sequence coverage obtained for three different recombinant proteins. Additionally, when assessing the μIMER digestion efficiency for complex proteome samples, it delivered a 144-fold faster and significantly more efficient protein digestion compared to 24 h for bulk digestion. The 3D-printed μIMER withstands remarkably high pressures above 130 bar and retains its activity for several weeks. This versatile platform will enable researchers to produce tailored polymer-based enzyme reactors for various applications in analytical chemistry and beyond.

## Introduction

Apart from rapid prototyping, 3D printing has become a viable method for manufacturing functional high-performance tools for research and development. Many exciting applications of additively manufactured devices in analytical chemistry have been presented in recent years.^1−6^ In contrast to the most widely used fused deposition modeling 3D printing,^2^ stereolithography (SLA) can produce airtight and watertight prints, holding enclosed and complex fluidic structures on the microscale.^7^ Despite the outstanding performance of SLA 3D printing of photocurable methyl methacrylate resin for the fabrication of polymethyl methacrylate microfluidic chips, an open port mass spectrometry (MS) interface^8^ as well as a polymer multi-array electrospray emitter^9^ are the only true online electrospray ionization (ESI)-MS applications where analyte solutions come in direct contact with the SLA prints. This is not surprising since residual uncured oligomers and additives tend to leach when in contact with aqueous and particularly organic solvents. Naturally, such contaminants are readily detected using a mass spectrometer and greatly interfere with the detection of low-abundant analytes. It was shown that rinsing the prints with pure ethanol or UV post-curing of clear resin SLA prints significantly reduced leaching and therefore enhanced the biocompatibility of 3D prints.^10^ Compounds leaching from the prints upon contact with aqueous buffers were analyzed by gas chromatography–MS and hypothesized to be residual monomers and short-length polymers.^11^

Alternatively, a range of thiol-ene UV-cured resin (TE)-derived monolithic chips coupled to MS were presented for solid-phase extraction,^12^ online protein digestion,^13^ or protein hydrogen–deuterium-exchange experiments (HDX) experiments.^14^ While proving highly effective for their individual tasks, these chips were molded in multi-step processes and did not support pressures beyond 500 psi (approx. 35 bar). Their pressure tolerance was nonetheless superior to that of typical polydimethylsiloxane (PDMS) chips. PDMS is the most prominently used material in microfluidics for modeling open fluidic channels.^15−17^ PDMS molds offer high resolution and water tightness but do not withstand high pressures. Typically, such molded chips are bonded to a plastic or glass support for sealing the channels. Leakage most likely occurs at the interconnections or via delamination from the support, typically at around 100 psi (approx. 7 bar).^18−21^ Hence, neither PDMS nor other molded resin chips withstand back pressures that typically occur in particle-packed microcolumns. Among other advantages, microparticle supports offer large surface areas that especially favor applications such as immobilized enzyme reactors (IMERs). For instance, protease IMERs are powerful tools for fast and easy sample preparation in bottom-up protein or proteome MS analysis. Protease IMERs are predominately flow-through devices, which are often intended for operation in consecutive digestion, trapping, and desalting steps with subsequent liquid chromatography (LC)-MS/MS analysis. Their numerous advantages over conventional digestion are well discussed in the corresponding literature.^22−29^ Several open tubular enzyme reactors^30−32^ or microfluidic chips with proteases immobilized on their inner wall^33,34^ have been developed. However, solid particle-based IMERs proved to be superior due to their dramatically larger surface areas and thus greater enzyme immobilization capacities.^35^ A large variety of particle-based^25,36−43^ or monolithic^13,44−50^ support materials has been utilized for this purpose.

Our goal was to develop a cost-effective and flexible platform for rapid peptic digestions in the context of protein HDX. In need of readily customizable, microbore as well as high-pressure-resistant column housings, we applied high resolution SLA 3D printing. The prints were rendered fit for application in MS by implementing a comprehensive washing and curing protocol. For protease immobilization, the benefits of carboxylated monodisperse polystyrene (PS) particles were exploited to produce highly effective pepsin μIMERs (here referred to as IMERs). The 3D-printed IMERs were designed to (i) be operated in an online LC–MS/MS setup in a plug and play fashion, (ii) withstand pressures above 100 bar without leaking, (iii) enable temperature monitoring, and (iv) retain their activity for several weeks. To assess the performance of the IMERs, we analyzed both recombinantly produced proteins and complex proteome samples. For all studied proteins, 100% sequence coverage was achieved, often exceeding 100 unique identifiable peptides of an average length below 12 amino acid residues. For complex proteome digestion, the IMER provided dramatically faster and significantly more efficient digestion compared to bulk digestion. This highlights the outstanding performance and robustness of the 3D-printed IMER as the first of its kind stereolithographically produced device for MS.

## Experimental Section

### Chemicals and Reagents

See the Supporting Information.

#### Microcolumn Manufacturing and SLA 3D Printing

The microcolumn chip was designed using an Autodesk Inventor 2020 (San Rafael, CA, U.S.). Inventor files were submitted for 3D printing via the manufacturer’s software PreForm V3.10.2 (Formlabs, Somerville, MA, U.S.). Printing was performed using standard *Clear V4* resin with a *Form3* low-force stereolithography printing unit (Formlabs). The layer thickness was set to 25 μm, and supporting structures were computed by the software *PreForm* and adapted manually. Fully open channels were obtained at a 45° angle tilt of the bore relative to the build platform and in parallel to the *Y*-plane. Post printing, the columns were rinsed for 30 min with tripropylene glycol monomethyl ether in a *FormWash* unit (Formlabs). Upon removing from the build platform, the connecting ports and threads were rinsed with fresh isopropyl alcohol (IPA). After a short drying period, both connecting ports were equipped with 1/16″ tubing and tightened with flangeless fittings and ferrules (Upchurch Scientific, Oak Harbor, WA, U.S.). The column was flushed at 50 μL·min^–1^ using a syringe pump (Harvard Apparatus, Holliston, MA, U.S.) with IPA, methanol, or acetone for at least 15 min. After disconnecting the tubing, the residual solvent was removed from the column using a microsyringe. The prints were allowed to dry for 1 h at room temperature. Finally, the prints were photocured for 180 min at 35 °C in a Form Cure (LED, 405 nm) oven (Formlabs).

#### Column Packing and Pepsin Immobilization

A 1/16″ PEEK capillary (130 μm I.D.) was connected to the column outlet using a flangeless 1/4″-28 PEEK fitting and ferrule. To retain the packing material, a 2 mm round hydrophilic filter membrane was clamped between the ferrule and the head of the column. The membrane (1.2 μm *Minisart* syringe filters; Sartorius, Goettingen, Germany) was cut to size using a 2 mm punch. Polybead carboxylated 3 μm polystyrene (PS) microspheres (2.6% solid particles in water and traces of the proprietary surfactant) were used for column packing. 125 μL of the microsphere suspension (equivalent to 3.2 mg of polystyrene microparticles) was loaded onto a loop of 1/16″ tubing, which was attached to the inlet of the microcolumn. The column was packed at a flow rate of 10 μL·min^–1^ for 18 min. During this process, the back pressure of the column increased to 80 bar. The pepsin coupling procedure was optimized for protein concentration, flow rate, and coupling time. The following protocol turned out to be most effective. 200 μL of the coupling solution [10 mg·mL^–1^ 1-ethyl-3-(3-dimethylaminopropyl)carbodiimide (EDAC) in the coupling buffer] was pumped through the packed column for 20 min at a flow rate of 10 μL·min^–1^. Subsequently, 200 μL of the freshly prepared pepsin solution (2.5 mg·mL^–1^ pepsin in the coupling buffer) was pumped through the packed column at 4 μL·min^–1^. The flow through was collected and used for determining the coupling efficiency by UV/vis spectroscopy. The column was finally flushed with 0.2% FA for 15 min and stored at 4 °C until use.

#### LC–MS Peptide Mapping

A Switchos II microcolumn switching module (LC Packings, Sunnyvale, CA, U.S.) equipped with two multi-port valves along with a 130 μm I.D. PEEK tubing was used for the online LC–MS setup. The loading pump delivered a flow of 0.2% FA in H_2_O throughout the online digestion, trapping, and desalting steps (8 min in total). Sample proteins were introduced via a 10 μL sample loop. Peptides were trapped on a SecurityGuard 4.0 mm I.D. × 3.0 mm C18 PreColumn (Phenomenex, Torrence, CA, U.S.). Separation was performed using a GromSil C18 HPLC column, 2.0 × 60 mm, 3 μm, 100 Å (Grom, Rottenburg, Germany) by 0.2% FA in H_2_O (mobile phase A) with 10 min gradient 5.0–50% mobile phase B (0.2% FA in ACN) plus 2 min at 50% B. The gradient was delivered using an LC20-AD HPLC system (Shimadzu, Kyoto, Japan) at 0.5 mL·min^–1^. This setup was coupled to a Thermo Scientific QExactive mass spectrometer (San Jose, CA, U.S), operated in the data-dependent acquisition (DDA) mode using top5 HCD fragmentation. For detailed MS settings and peptide identification, see the Supporting Information.

### RocC Bulk Digestion

See the Supporting Information.

#### Testing for Leachables

Direct infusion MS experiments were performed to assess the suitability of the 3D-printed chips for coupling with ESI mass spectrometry. Empty channel chips were coupled to a LTQ Orbitrap XL mass spectrometer, equipped with a standard ESI source, and flushed with 0.2% aqueous FA at a flow of 5 μL·min^–1^. Leachable testing was performed on uncured chips, and chips which were washed with either isopropanol, acetone, or methanol for at least 15 min prior to the photocuring step. The efficacy of the washing step was determined by injections of 5 μL of a 10 μM cytochrome c solution via a sample loop.

#### Protein Extraction from Cell Culture and IMER Digestion

HEK293T cells were grown to full confluency at 37 °C and in a 5% CO_2_ atmosphere in DMEM with 4.5 g·L^–1^ glucose. Proteins were extracted by lysing the cells with 8 M urea in 100 mM ammonium bicarbonate (pH 8.5). Cells were scraped, resuspended, and transferred to a tube. Extracted protein amounts were determined using a colorimetric bichinonic acid assay (Thermo Fisher Scientific, Dreieich, Germany). Before digestion, proteins were reduced by addition of 10 mM dithiothreitol (DTT) and alkylated with 20 mM iodoacetamide. After alkylation, DTT was added to stop overalkylation. Proteome solutions of 0.125, 0.25, 0.5, and 1 mg·mL^–1^ protein in 3 M urea, 100 mM NH_4_HCO_3_, and 3% FA were prepared. Injections of 20 μL were digested online at 10 μL·min^–1^ for 10 min at room temperature, starting with three blank injections. The flow through was collected in LoBind Eppendorf vials. Samples were frozen at −80 °C and lyophilized in a SpeedVac centrifuge (Thermo Scientific).

#### Gel Electrophoresis and Coomassie Staining

Proteome digests were reconstituted in 20 μL of 100 mM NH_4_HCO_3_ buffer, and 5 μL of Laemmli buffer was added. Sodium dodecyl sulfate-polyacrylamide gel electrophoresis (SDS-PAGE) was performed by loading the samples on a gel containing 10% acrylamide. The gels were run at 90 V for 110 min. Afterward, gels were stained with SimplyBlue SafeStain (Thermo Fisher Scientific, Dreieich, Germany) for 1 h and washed twice with water, once for 1 h, and once overnight.

#### UHPLC–MS Proteome Analysis

Dried peptides were dissolved in 20 μL of 0.1% FA, and 10 μL was injected onto the trapping column (nanoEase M/Z Symmetry C18 Trap Column, 100 Å, 5 μm, 180 μm × 20 mm, Waters, Manchester, UK) of a nano-UPLC system (UltiMate 3000 RSLCnano System, Thermo Fisher Scientific, Bremen, Germany) with a flow rate of 30 μL/min using 5% B (A: 0.1% FA in H_2_O, B: 80% ACN, 0.1% FA in H_2_O). Peptides were eluted and separated on a separation column (nanoEase M/Z Peptide BEH C18 Column, 130 Å, 1.7 μm, 75 μm × 250 mm, Waters, Manchester, UK) using a gradient from 5 to 27.5% B in 105 min, followed by 27.5–40% B in 10 min at a flow rate of 300 nl·min^-1^. The nano-UPLC system was connected to an orbitrap mass spectrometer (Orbitrap Fusion Lumos, Thermo Fisher Scientific, San Jose, CA, USA) via a nano-ESI source. The spray was generated from a steel emitter (Fisher Scientific, Dreieich, Germany) at a voltage of 1850 V. MS/MS measurements were carried out in the DDA mode using a normalized HCD collision energy of 30%. Every 3 s, one MS scan was performed over an *m*/*z* range from 375 to 1500, with a resolution of 120,000 at *m*/*z* 200 (maximum injection time = 50 ms, AGC target = 2 × 10^5^). MS/MS spectra were recorded in the orbitrap with a resolution of 15,000 at *m*/*z* 200 (maximum injection time = 54 ms, maximum AGC target = 5 × 10^4^, intensity threshold: 2.5 × 10^4^, first *m*/*z*: 110), a quadrupole isolation width of 1.6 Da, and an exclusion time of 60 s. For peptide and protein identification and quantification, LC–MS raw data were processed with MaxQuant (version 1.6.17.0). For identification, MS/MS spectra were searched with the Andromeda search engine against a human swiss-prot database (20,431 entries, www.uniprot.org) and a contaminant database (298 entries). The searches were performed using the following parameters: the precursor mass tolerance was set to 20 ppm for a first peptide search, and the main search was performed with a tolerance of 4.5 ppm. For MS/MS spectra, a fragment mass tolerance of 20 ppm was used. Enzyme specificity was set to unspecific, and the following modifications were considered: carbamidomethylation on cysteine residues as a fixed modification and oxidation of methionine residues as a variable modification. Peptides and proteins were identified with an FDR of 1%. Proteins were kept as correctly identified if at least one unique peptide was identified. Peptides and proteins were quantified with the MaxLFQ algorithm, considering only unique peptides and a minimum ratio count of 1. Bioinformatics data processing (log2-transformation, normalization), statistical analysis (two-sided Student’s *T*-test using a permutation-based FDR with an adj. *p*-value cutoff of 0.05), and data visualization were performed with Perseus (version 1.6.7.0).

## Results and Discussion

### IMER Fabrication and Characterization

**Figure 1:**
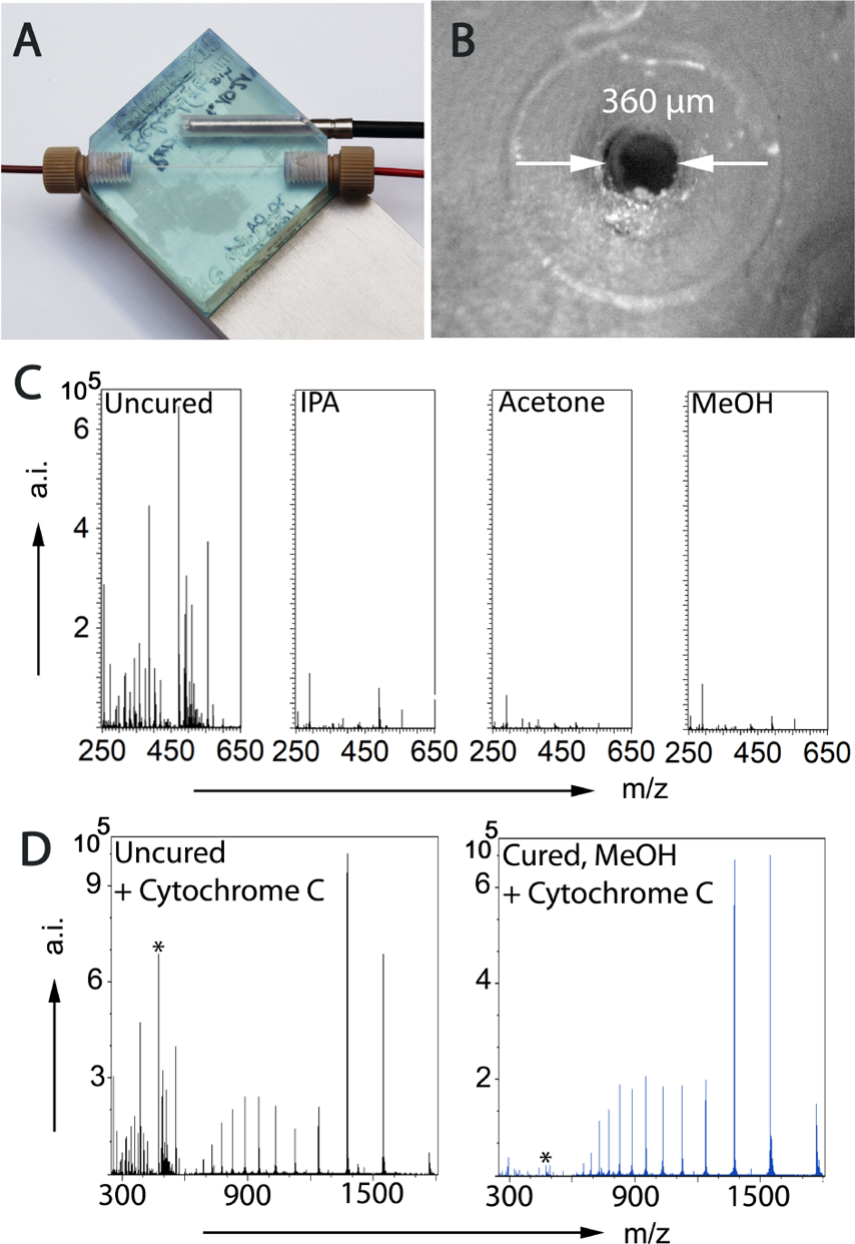
Photographical image of the transparent SLA-printed cartridge equipped with a temperature probe (A); microscopy image of the open bore surrounded by the imprint of the shape and diameter of an 1/16″ PEEK capillary (B); positive ion mode mass spectra for the determination of leachables from SLA 3D prints were obtained by direct coupling of the chips to MS and flushing with 0.2% aqueous FA at a flow rate of 5 μL·min^–1^: uncured chip (C; left); chips washed with isopropanol, acetone, or methanol for at least 15 min and cured with 405 nm blue light at 35 °C for 3 h (C; center left to right); positive ion mode mass spectra obtained by direct coupling of the chips to MS, flushing with 0.2% aqueous FA at a flow rate of 5 μL·min^–1^ and injecting 5 μL of a 10 μM cytochrome c solution via a sample loop. The intensity of the signal related to the leachable UDMA at *m*/*z* = 471.2685 (marked by an asterisk) is 64,000 a.i. for the uncured chip (D; left) and 200 a.i. for the cured (D; right). See the Supporting Information for MS settings.

The low-force SLA printer *Form3* offers an *XY*-lateral resolution of 25 μm, while the *Z*-resolution, that is, layer thickness, can be adjusted to 25, 50, or 100 μm. However, the direct SLA 3D printing of bores below 0.5 mm inner diameter is very challenging in the case of the targeted diameter to length ratios (Figure 1A,B). In the case of the *Form3* printer, narrow open channels could only be obtained at a 45° angle tilt of the bore relative to the build platform, while the bore is parallel to the *Y*-plane. Moreover, the lower limit for the bore diameter was determined to be 0.5 mm in the CAD drawing, which reproducibly resulted in an effective column diameter of approx. 360 μm (see the Supporting Information). We hypothesize two factors to be responsible for this bore narrowing: (i) the laser light penetrating the transparent resin must have polymerized additional material (*Z*-overcuring) and (ii) the slight expansion of cured resin upon post-curing or during the polymerization process. Given the true column dimensions of 360 μm I.D. × 30 mm, a column volume of 3.06 μL was determined. Additionally, the formation of a 1/16″ diameter polymer crater surrounding the microbore was observed (Figure 1B). Apparently, the force inflicted by thoroughly tightening the fitting was sufficient to imprint the PEEK capillary’s shape into the rigid polymer to create a tight seal. This self-sealing capacity enabled the operation of the column at high pressures without leaking or pressure drops.

### Testing for Leachables

Direct infusion MS experiments were performed to assess the suitability of the 3D-printed chips (Figure 1A) for coupling with ESI mass spectrometry. Empty channel chips (Figure 1B) were coupled to an LTQ Orbitrap XL mass spectrometer, equipped with a standard ESI source, and flushed with 0.2% aqueous FA at a flow of 5 μL·min^–1^. Leachable testing was performed on uncured chips, and chips which were washed with isopropanol, acetone, or methanol (Figure 1C) for at least 15 min prior to the photocuring step. The efficacy of the washing step was determined by injections of 5 μL of a 10 μM cytochrome c solution via a sample loop. As can be seen in Figure 1D, the mass spectra obtained from the eluate of uncured chips showed prominent signals in the range < *m*/*z* = 600, which corresponded to contaminants leaching from the SLA print. It is also evident from Figure 1D that these compounds will strongly interfere with any mass spectrometric detection of compounds in a mass range which is vital for peptide analysis. Further MS/MS analysis of the most abundant signal at *m*/*z* = 471.2685 (*z* = 1) plus the respective ammonium, sodium, and potassium adducts revealed characteristic fragmentation patterns for methacrylate compounds (see Figure S1, Table S1, and Scheme S1). Based on the computed elemental composition and characteristic fragment ions, the signals were assigned to the compound urethane dimethacrylate UDMA, a common crosslinking agent in methacrylate resins. Clearly, the compounds leaching from the SLA prints are mostly uncured oligomers or crosslinking agents and degradation products thereof. However, all three post-print protocols drastically decreased the intensity of leachables to an acceptable level with acetone and methanol performing the best. In both cases, the ion count of the UDMA sodium adduct declined from 30,000 pre-curing to below 1000, while the protonated UDMA species was barely detectable at 200 counts. Washing with acetone, however, compromised the structural integrity of the capillary. This was evident by the capillary wall turning from fully transparent to a cloudy shade. Similar effects of acetone and acetonitrile on SLA prints have been reported.^51^ Hence, methanol-treated chips were chosen for further experiments.

### Online/In Situ Enzyme Immobilization

**Figure 2:**
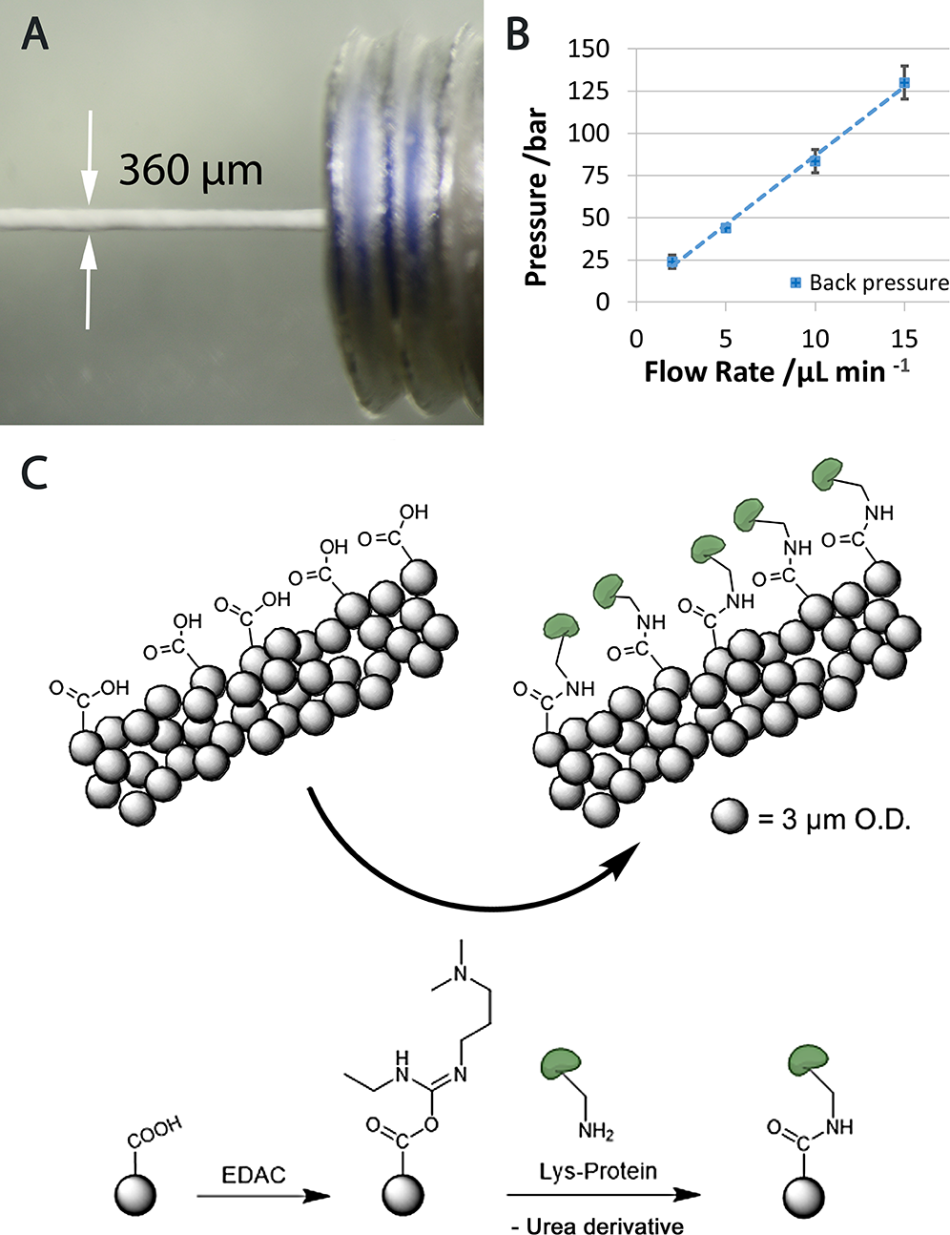
IMER fabrication and characterization: photographical image of the 360 μm diameter column, packed with solid 3 μm PS particles (A); linear correlation of flow rate and back pressure on the 30 mm × 360 μm I.D. packed microcolumn (B); pepsin coupling utilizing EDAC activation (C).

Despite the used coupling kit and microspheres being intended for coupling and operation in bulk solution, we chose an online approach after some iterations. Online coupling is more difficult to perform from a technical perspective but holds several advantages. Most importantly, lengthy and repetitive washing, that is, centrifugation steps, can be avoided in flow-through operation. Furthermore, using the manufacturer’s coupling protocol was not possible since it involves washing steps of the enzyme-loaded particles at pH 8 (wash/storage buffer), eventually leading to irreversible inactivation of pepsin. During the packing process, pressures above 80 bar were observed without leaking (Figure 2A,B). After column packing, the modified polystyrene surface was activated with EDAC. Finally, the protease was immobilized by flushing the activated column with 500 μg of pepsin dissolved in 200 μL of the coupling buffer (Figure 2C). The protease loading was determined using a UV/vis NanoPhotometer Classic (Implen, Munich, Germany). Online pepsin coupling was successfully employed as described in the Experimental Section, yielding a total coupling of 102 ± 3 μg pepsin on 3.2 mg of the solid support material. This translates to 135% of the manufacturer’s claim for expected protein loading by offline coupling (300 μg of IgG on 12.5 mg of particles). The key parameters and characteristics regarding the column and the IMER are summarized in Supporting Information Table S2.

### Quantitative Assessment of IMER Performance

**Figure 3:**
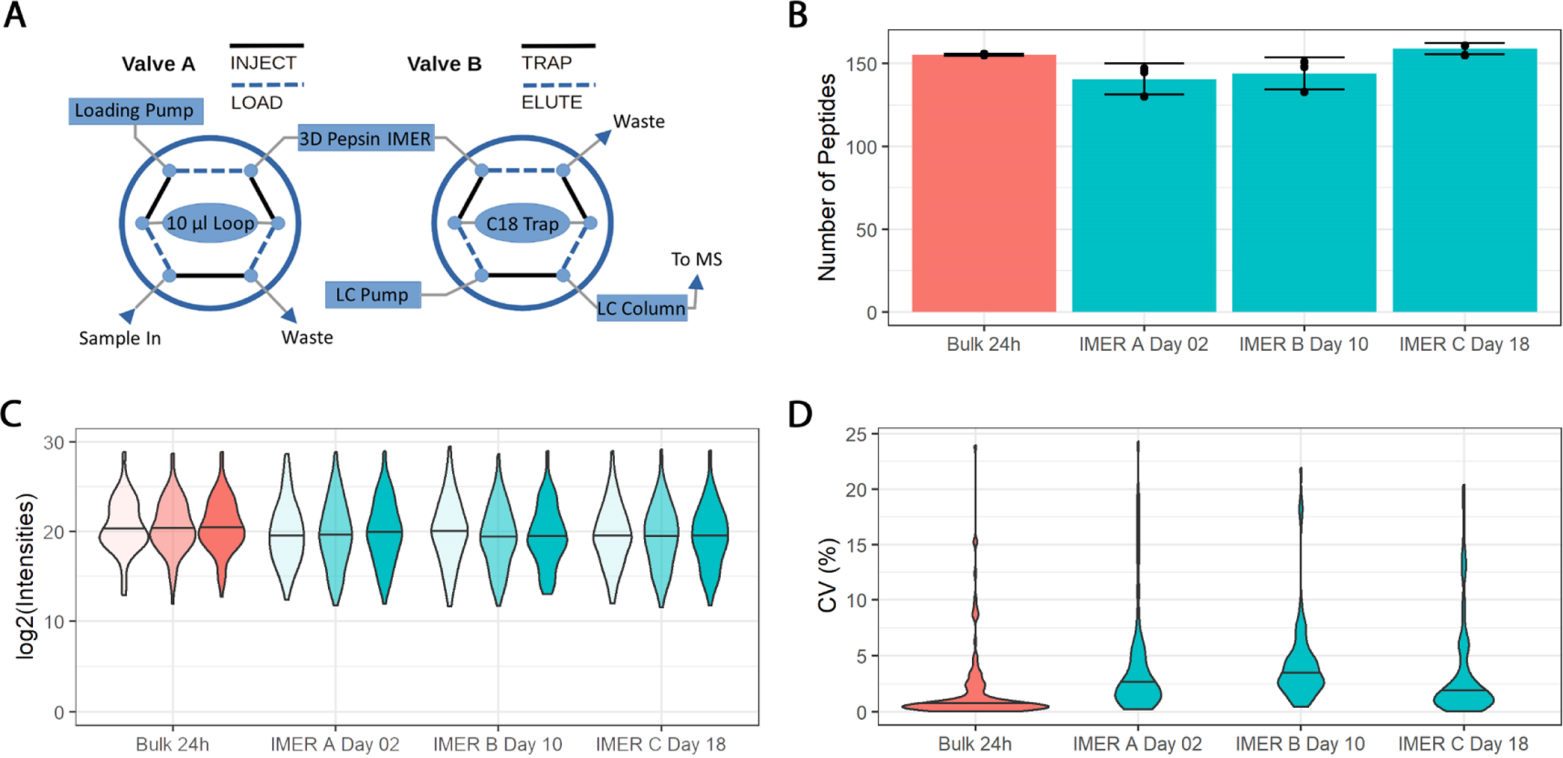
Experimental setup for the online protein analysis using two multi-port valves for online digestion, desalting, and LC–MS analysis of proteins (A); quantitative IMER characterization based on the peptic digestion of in-house recombinant protein RocC: total number of peptide hits and number of unique identified peptides detected in bulk digestions (24 h) and IMER online digestions performed on three different days; the error bars represent the mean plus/minus standard deviation, *N* = 3 (B); violin plot showing the distribution of intensities for all quantified peptides. Three replicates are shown for each IMER or the bulk (C); violin plots showing distribution of CV calculated for peptide intensities in *N* = 3 replicates (D). See Supporting Information Figure S4 for volcano plots.

For online protein analysis, the IMER was implemented in the LC–MS setup as shown in Figure 3A. The IMER was utilized to obtain sequence confirmation by peptide mapping of three different in-house recombinantly produced proteins. As the first protein studied, 0.46 μg of samples of the RNA chaperone FinO-domain RocC (produced as described by Eidelpes et al.^52^) in 0.8% FA and 1 mM NH_4_HCO_3_ was digested online at 10 μL·min^–1^, trapped on a 4.0 × 3.0 mm C18 column and desalted (8 min in total). The average pressure during this process was 85 bar. This was followed by data-dependent LC–MS/MS analysis in a 10 min gradient (for LC and MS/MS settings, see the Experimental Section). The experiments were performed in three consecutive runs.

In spite of the extremely short digestion time, the IMER was able to effectively digest the sample proteins and deliver a substantial number of short-length peptides for sequence analysis and confirmation. Remarkably, we were able to produce three different IMERs (A, B, and C) in intervals of at least 1 month, which showed comparable performance and properties. For all IMERs, a comparable number of peptic RocC peptides were identified (IMER A: 141 ± 9 peptides, IMER B: 144 ± 10 peptides, IMER C: 159 ± 3 peptides, Figure 3B) and no significant differences in overall intensities were observed (Figure 3C). See Supporting Information Figure S2 for the peptide map and S4 for volcano plots. For both IMER and bulk digestion, the quantified peptides have a coefficient of variation (CV) lower than 25% (Figure 3D). Each of the IMERs was able to rival the digestion performance of the 24 h bulk reaction, highlighting the quality and reproducibility of the presented protocol. Minor differences can be attributed to varying MS instrument performance, given the time spans between the experiments. Importantly, all IMERs showed high run-to-run as well as IMER-to-IMER reproducibility (Figures 3B–D and S4). All experiments yielded 100% sequence coverage for RocC with over 100 identified peptides for each experiment. Therefore, we conclude that the performance of the IMER is highly reproducible. Complete sequence coverage of the recombinant sample protein was achieved within an experiment runtime of 20 min per run, which included protein digestion, desalting, and LC–MS analysis. The IMERs therefore enable ultra-fast peptide mapping in less than 1.4% of the experiment time of the bulk digest approach.

The above-mentioned online digestion experiments were repeated with different IMERs at different lifetimes up to 29 days to assess the longevity of the surface bound protease. Even after storage for over 4 weeks at 4 °C, the IMER delivered high and reproducible performance, resulting in 100% sequence coverage for the studies the RocC protein, only showing a slight loss of intensity (for details, see Figure S3).

IMER online digestion and hyphenated LC–MS analysis were further performed for two additional in-house produced recombinant proteins: the allergens Mald1.0201 and Act c 8. Here, peptide annotation was performed using the freely available software MSstudio.^53^ For Mal d 1.0201, 0.5 μg injections were performed at either 5 μL·min^–1^ or 10 μL·min^–1^. The lower flow rate naturally resulted in less back pressure at 45 bar compared to 85–90 bar at 10 μL·min^–1^. The low flow rate run yielded 110 peptide hits, while the run at a regular flow rate resulted in 96 peptide hits with 100% sequence coverage in both cases (Figure S6). The IMER was further tested at 15 μL·min^–1^, which resulted in 135 bar back pressure. Again, no leakage was observed as the pressure remained constant for several minutes. However, such high flow rates were not used further so as to ensure greater longevity and prevent any column bed damage. The manufacturer does not state any pressure limit for the used polystyrene microspheres as they are not intended for high-pressure applications. Therefore, 10 μL·min^–1^ was identified as a suitable operational flow rate. The observed back pressures at given flow rates are comparable to those of 3 μm nonporous particle HPLC columns of similar lengths and diameters.

The foodborne allergen Act c 8 was recombinantly produced and purified as described by Zeindl and Tollinger.^54^ The online digestion and desalting setup allowed for the direct analysis of the protein in its storing buffer. 2.5 μg of Act c 8 samples in 3 M guanidine hydrochloride was injected in three consecutive runs for online digestion at ambient temperature on day 16 of the IMER lifetime. The addition of the chaotropic agent to the sample buffer resulted in thorough digestion and 100% sequence coverage with 119.3 ± 9.2 peptide hits per run. A total of 115 of the peptides were present in at least 2 of 3 runs and therefore labeled as unique identifiable (Figure S7). We conclude that the 3D-printed microcolumn IMER can safely be used with high concentrations of chaotropic agents. See the Supporting Information for additional details. The extremely fast analysis capability enabled by IMER online digestion is highly favorable when supporting in-house protein biochemists in the development of protein expression protocols and downstream processes. The IMER allows us to obtain information on protein identity and primary sequence integrity in less than 30 min.

#### Complex Proteome Digestion Efficiency

**Figure 4:**
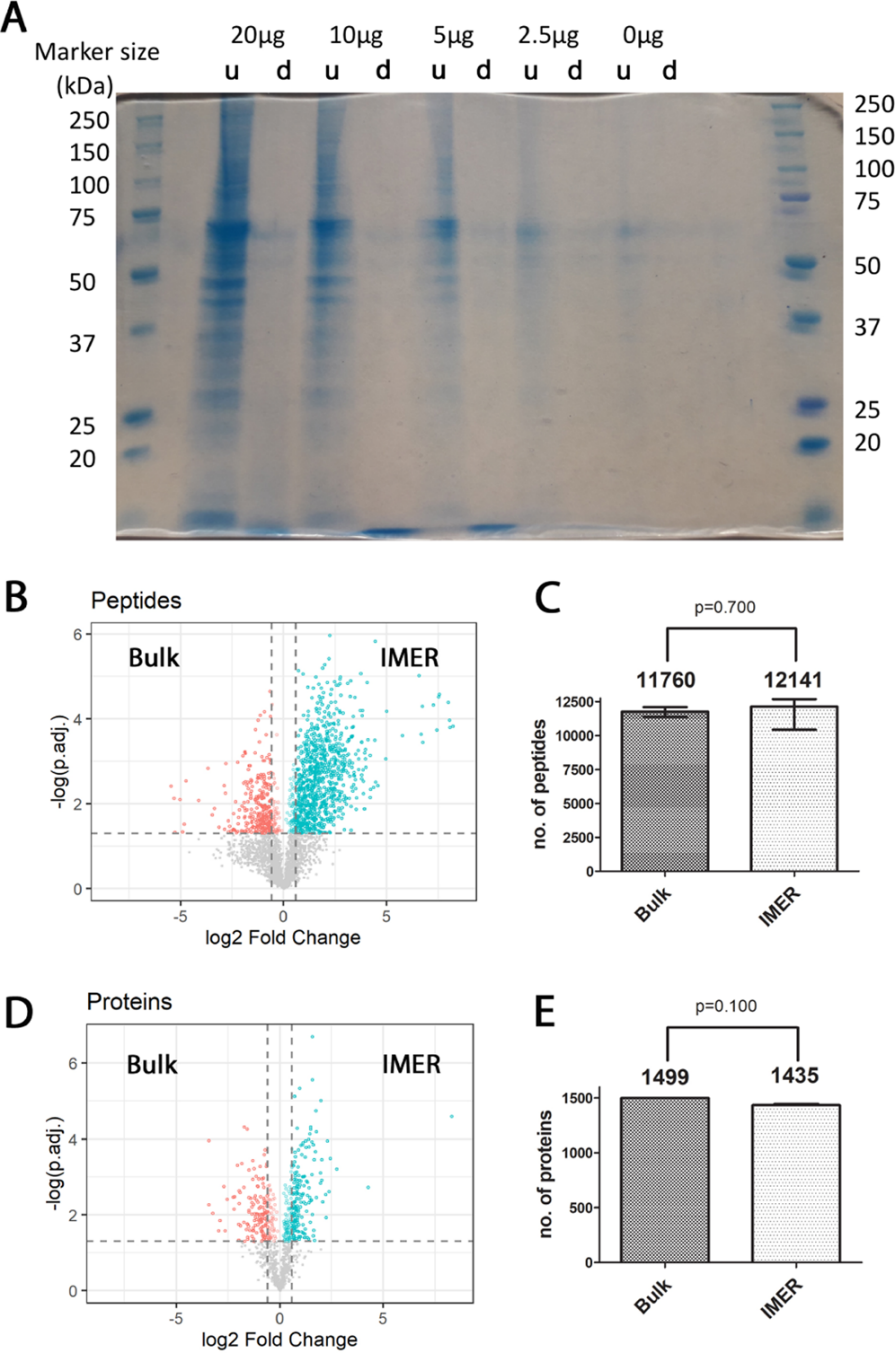
Quantitative analysis of digestion efficiency between bulk and IMER digestion of the complex proteome. (A): SDS-PAGE of the IMER-undigested proteome extract (u) and digested proteome extract (d). Very left and very right: protein standard. The different sample amounts loaded on the SDS-PAGE system are indicated accordingly. (B): Volcano plots of quantified peptides comparing bulk digestion and IMER digestion. (C): Bar graphs (median with standard deviation) showing the number of identified peptides after bulk and IMER digestion. (D): Volcano plots of quantified proteins comparing bulk digestion and IMER digestion. (E): Bar graphs (median with standard deviation) showing the number of identified proteins after bulk and IMER digestion. (C,E): Statistical analyses were performed using the two-tailed Mann–Whitney test. (B,D): Adjusted *p*-value of ≤0.05 was used as a threshold for statistical significance. Peptides and proteins showing significant higher amounts in bulk or IMER digestion are colored in red (bulk) or blue (IMER). Analytes with a fold-change of ≤1.5 are represented as red- or blue-colored semi-transparent dots, and analytes with fold-change of ≥1.5 are represented as red- or blue-colored dots. *N* = 3 independent experiments.

To examine the performance of the IMER for global proteome analysis, we investigated the enzymatic digestion efficiency of the IMER for a complex proteome sample. Here, different amounts of a proteome extract isolated from human embryonic kidney 293T cells were loaded onto the pepsin IMER. The eluents of the IMER were collected and evaporated. The dried eluents were dissolved in 20 μL of 100 mM ammonium bicarbonate (pH 8.3); 5 μL of 5× Laemmli buffer was added, and the samples were loaded onto an SDS-PAGE system. As a control, the same amounts of nondigested proteome extracts were loaded onto the SDS-PAGE system. The comparison of SDS-PAGE traces of the undigested proteome and IMER-digested samples showed that IMER efficiently digested proteomes up to 20 μg (Figure 4A). The trace of the IMER-digested proteome amount of 20 μg showed some weak bands. This indicates that the maximum amount of proteome to be used for the IMER is about 20 μg.

To compare the performance of proteome digestion between conventional bulk digestion and IMER, we digested 5 μg of the proteome extract with pepsin (protein/protease ratio 100:1) overnight and loaded 5 μg of the proteome extract onto the IMER. Both overnight digested samples and eluents of the IMER were dried in a SpeedVac centrifuge. Dried samples were dissolved in 20 and 10 μL thereof were subjected to LC–MS/MS analysis for label-free quantification. No significant differences were observed between bulk digestion and online IMER digestion in terms of the total number of identified peptides (nIMER = 12,141 ± 1171; nBULK = 11,760 ± 363; *p* = 0.700, Figure 4B) and identified proteins (nIMER = 1435 ± 60; nBULK = 1499 ± 15; *p* = 0.100, Figure 4C). A quantitative comparison at the protein level showed that a larger number of proteins could be quantified with a significantly higher amount in general for IMER-digested samples: (nIMER = 221, nBULK = 156) and at least by a factor of 1.5 (nIMER = 150, nBULK = 114) (Figure 4D).

To compare the performance of bulk and IMER digestion for the analysis of complex proteomes, the quantitative comparison at the peptide level is much more relevant because the peptides were the product of pepsin digestion, and the digestion efficiency can be compared based on the quantity of peptides generated. The quantitative analysis at the peptide level showed that 324 peptides were detected in significantly higher quantities for the bulk digestion, whereas 945 peptides were detected in significantly higher quantities in samples generated during IMER digestion (Figure 4E). It is worth mentioning that 93% of these 945 peptides, that is, 879 peptides, showed at least 1.5-fold higher amount in IMER than in bulk digestion, where 273 peptides showed at least 1.5-fold higher amount. These results show that IMER allows a 144-fold faster (10 min for IMER, 24 h for bulk digestion) and significantly more efficient digestion of complex proteomes in the lower-microgram range compared to conventional bulk digestion, representing a clear advance in proteome profiling.

## Conclusions

Using high-resolution additive manufacturing, it was possible to produce microfluidic chips holding an enclosed 360 μm I.D. microbore column with an aspect ratio close to 100:1. Leachables derived from the 3D prints were identified as the crosslinking agent UDMA and degradation products thereof. The leachable derived sample contamination was drastically reduced by implementing a comprehensive washing and curing protocol. Thereafter, SLA prints were suitable for hyphenation with MS and further utilized to create microscale pepsin-immobilized enzyme reactors. The IMER proved highly effective for thorough online digestion of different recombinantly produced proteins, was implemented in an online setup, and (i) showed outstanding structural integrity, withstanding astonishingly high pressures above 130 bar without leaking, (ii) achieved 100% sequence coverage for all studied proteins, (iii) displayed excellent performance even after 4 weeks of storage, and (iv) was successfully used with 3 M guanidine hydrochloride and urea for improved protein denaturation. Furthermore, the IMER provided dramatically faster (144-fold) and significantly more efficient digestion of complex proteomes in the lower μg range. This IMER therefore represents an interesting and powerful technology for quantitative proteomics of the smallest sample amounts.

Apart from the pepsin IMER presented here, this protocol essentially represents a versatile platform for many more future applications. The presented protocol, relying on robust coupling chemistry and commercial products, can be expanded to virtually any protein of choice to create either different IMERs or other applications where protein immobilization is favorable, for example, assays for ligand screening. Using the developed washing and curing protocol, we expect to see many more exciting applications of SLA prints in the field of analytical chemistry. Given the unique versatility of 3D printing, some of these might include multi-bed reactors, composed of multiple columns of varying dimensions in a single chip or the incorporation of differently loaded particles in a single column. SLA 3D printing enables the direct manufacturing of such true 3D geometries. In this context, the use of SLA-printed components could be expanded to integrated multi-step systems for sample protein online reduction, deglycosylation, and digestion. These components will likely exhibit performances on par with milled glass chips but at much lower costs and faster lead times.

As high-resolution SLA printing is becoming more and more accessible, researchers are pushing its boundaries toward producing increasingly smaller and better resolved microfluidic designs. Hence, we anticipate a striking impact of SLA on the production and prototyping of polymer-based devices for custom-designed enzyme reactors. Given their highly favorable self-sealing capacity and excellent pressure resistance, SLA prints will greatly contribute to the evolving field of high-pressure microfluidics.

## Supporting Information Available

Identification of leachables by tandem MS, additional experimental information on peptide mapping and MS, peptide maps and volcano plots, and further IMER specifications (PDF)
