# Supplementary material for: 3D-Printed High-Pressure-Resistant Immobilized Enzyme Microreactor (μIMER) for Protein Analysis
Source: Anal Chem. 2022 Jun 9;94(24):8580–7. doi: 10.1021/acs.analchem.1c05232 (PMC9218953; doi:10.1021/acs.analchem.1c05232)
Supplement: Supplementary file 1 — ac1c05232_si_001.pdf [file ac1c05232_si_001.pdf]

Supporting Information

3D-Printed High-Pressure-Resistant Immobilized Enzyme Microreactor (μIMER) for Protein Analysis

Tobias Rainer,[a] Anna-Sophia Egger,[b] Ricarda Zeindl,[a] Martin Tollinger,[a] Marcel Kwiatkowski[b]* and Thomas Müller[a]*

[a] Institute of Organic Chemistry and Center for Molecular Biosciences (CMBI), Leopold-Franzens University Innsbruck, Austria

[b] Institute of Biochemistry and Center for Molecular Biosciences (CMBI), Leopold-Franzens University Innsbruck, Austria

*Corresponding authors:

Marcel Kwiatkowski – Email: Marcel.Kwiatkowski@uibk.ac.at

Thomas Müller – Email: Thomas.Mueller@uibk.ac.at

Table of content

1 Reagents and Chemicals S2

2 Direct Infusion Mass Spectrometry, Testing for Leachables S2

3 RocC Bulk Digest S3

4 Leachable Identification by MS/MS S4

5 IMER Technical Characteristics S6

6 Peptide Mapping LC-MS, Data Dependent Acquisition and Peptide Identification S8

7 Qualitative Assessment of IMER Performance S9

8 Quantitative Assessment of IMER Performance S11

9 Peptides Maps, Produced by Online IMER Digestion and LC-MS/MS S12

10 Assessing 3D Printing Reproducibility and Bore Integrity S14

11 References S15

1 Reagents and Chemicals

Clear V4 methyl methacrylate photocurable resin *(*Formlabs*)* and tripropylene glycol monomethyl ether (TPM) RS-01 resin cleaner were from 3Dee (Vienna, Austria). Polybead® carboxylated 3 µm polystyrene microspheres (2.86 µm average diameter with CV = 4%, lot number A794429) and PolyLink® protein coupling kit for COOH-microparticles were obtained from Polyscience Inc. (Warrington, PA, U.S.). The PolyLink® coupling kit includes: 1-ethyl-3-(3-dimethylaminopropyl) carbodiimide (EDAC), PolyLink coupling buffer (50 mM MES, pH 5.2, 0.05% Proclin® 300) and PolyLink wash/storage buffer (10 mM TRIS, pH 8.00, 0.05% BSA, 0.05% Proclin® 300). Formic acid (FA) puriss. ≥ 98% was from Merck (Darmstadt, Germany). Methanol HiPerSolv® ≥ 99.8%, was supplied by VWR (Randor, PA, U.S.). Acetone for HPLC, ≥ 99.8%, isopropyl alcohol (IPA), LiChrosolv® hypergrade ≥ 99.9%, pepsin from porcine gastric mucosa (P6887), lyophilized powder ≥ 3200 $U \cdot mg^{-1}$ and cytochrome c from equine heart (C7752), ≥ 95% were acquired from Sigma-Aldrich (St. Louis, MO, U.S.). Guanidine hydrochloride (GdnHCl) > 98% was from Carl Roth (Karlsruhe, Germany). Ammonium bicarbonate (Honeywell, Fluka™), a micro BCA™ assay kit and SimplyBlue™ SafeStain was aquired from Thermo Fisher Scientific (Dreireich, Germany). Dulbecco's Modified Eagle Medium was purchased from PAN Biotech (Aidenbach, Germany).

2 Direct Infusion Mass Spectrometry, Testing for Leachables

Preliminary direct infusion experiments were carried out on a LTQ Orbitrap XL mass spectrometer (Thermo Fisher, San Jose, CA, U.S.) equipped with a standard ESI ion source. The instrument was operated in positive ion mode at +3 kV spray voltage, 15 arbitrary units sheath gas pressure, 37 V capillary voltage, 200 °C capillary temperature and 100 V tube lens voltage. Full MS spectra were acquired for the mass range from m/z = 200 to 2,000. Data were acquired in continuous mode, averaging two scans at 500 ms max injection time, the resolution was set to 60,000 (at m/z = 200).

The 3D printed columns were connected to the microcolumn switching modul *Switchos II* (LC Packings, Sunnyvale; CA, U.S.) and the mass spectrometer respectively. The *Switchos* loading pump delivered a flow of 0.2% FA in H_2O at 5 $\mu L \cdot min^{-1}$. Samples of 10 µM cytochrome c were injected over a 5 µL stainless steel sample loop as described in the Results and Discussion section.

3 RocC Bulk Digest

For the comparison of the digestion efficacy between immobilized and free protease enzyme, a bulk digest was performed. A solution of 230 $\mu g \cdot mL^{-1}$ RocC in 5 mM NH_4HCO_3 was diluted 1:5 (v/v) with 1% FA to a final concentration of 46 $\mu g \cdot mL^{-1}$ in 0.8 % FA and aliquoted to three 100 µL portions. 2 µL of freshly prepared 100 $\mu g \cdot mL^{-1}$ pepsin in 1% FA were added to each vial. The mixtures were incubated at room temperature for 24 hours (Figure 3 and Supporting Figure S4) as well as for one hour (see Figures S2 and S3) and subsequently inactivated by heat shock to 95°C for 5 min. Digested samples were stored at - 80°C until analysis.

4 Leachable Identification by MS/MS

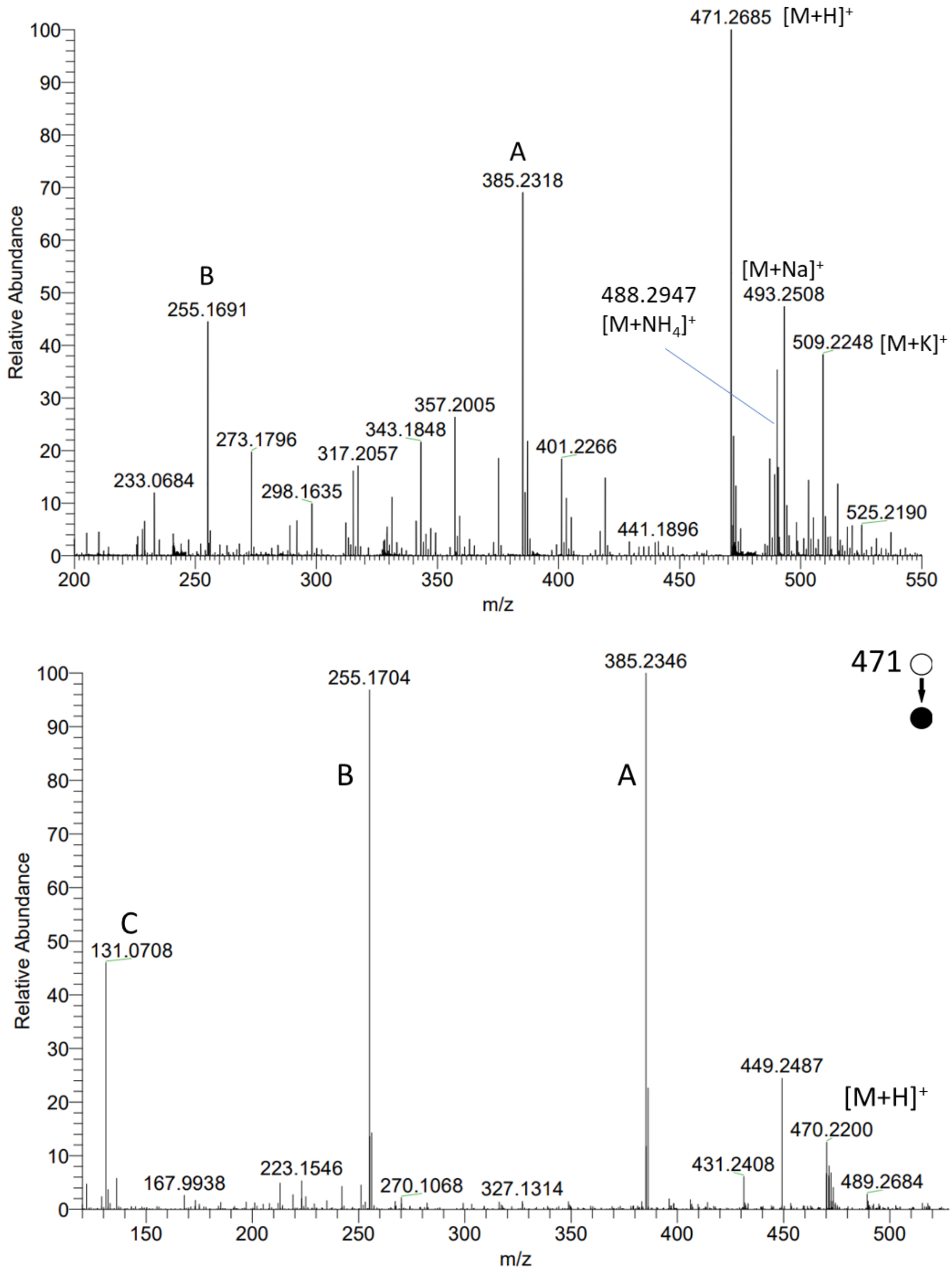

Supporting Figure S1. Top: Full MS (range m/z = 200 to 550) of 0.2% FA flow at 5 µL·mL^{-1} through an uncured microcolumn chip, directly coupled to the MS. Bottom: MS/MS spectrum of the 471 m/z precursor with a 27% normalized collision cell energy (HCD), characteristic fragments are annotated and listed in Supporting Table S1.

Supporting Table S1. Postulated ion and fragment annotation regarding the mass spectra in Figure S1.

ID	Structure	Chemical formula and theoretical m/z value
**$[M+H]^+$**	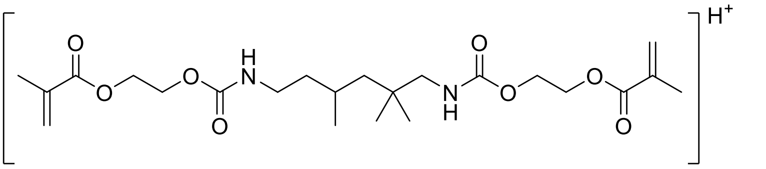	$C_{23}H_{39}N_2O_8^+$ m/z = 471.2701
**$[M+NH_4]^+$**	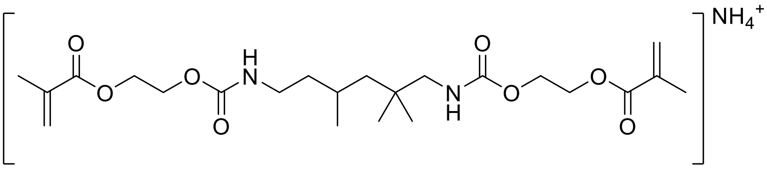	$C_{23}H_{42}N_3O_8^+$ m/z = 488.2966
**$[M+Na]^+$**	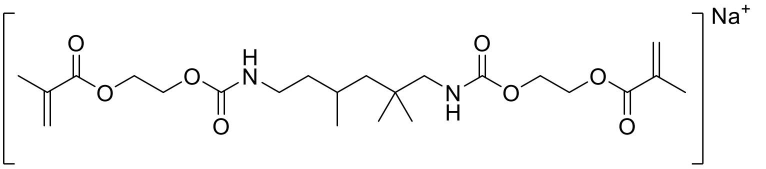	$C_{23}H_{38}N_2O_8Na^+$ m/z = 493.2520
**$[M+K]^+$**	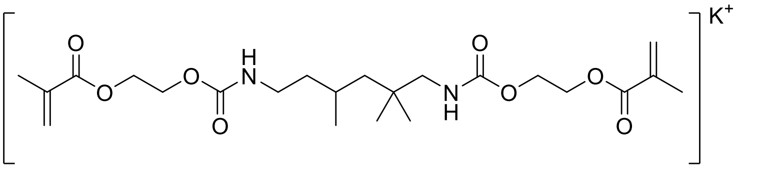	$C_{23}H_{38}N_2O_8K^+$ m/z = 509.2260
**A**	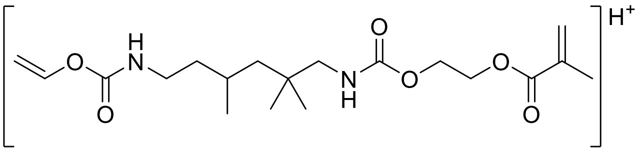	$C_{19}H_{33}N_2O_6^+$ m/z = 385.2333
**B**	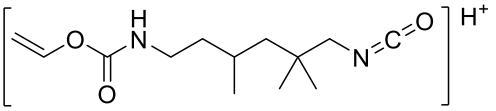	$C_{13}H_{23}N_2O_3^+$ m/z = 255.1703
**C**	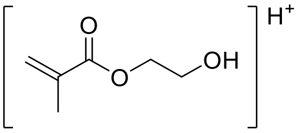	$C_6H_{11}O_3^+$ m/z = 131.0703

Supporting Scheme S1. Postulated fragmentation mechanism for the $[UDMA+H]^+$ precursor at m/z = 471. Characteristic fragmentation reactions for urethane compounds are discussed by Gies et al.[1]

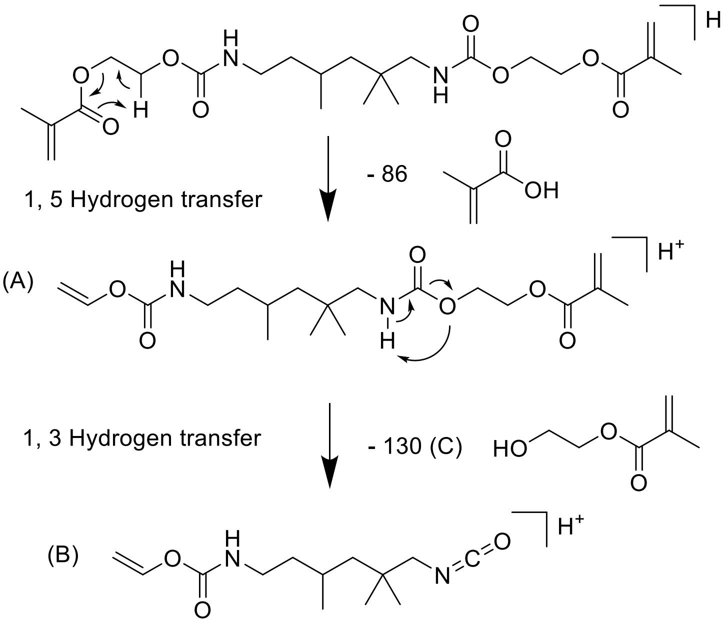

5 IMER Technical Characteristics

The IMER was characterized based on experimental parameters, UV spectroscopy and calculations using the manufacturer's technical data sheet as follows:

Supporting Table S2. Properties and characteristics of the manufactured microbore column and pepsin IMER.

Column Properties	
**Description**	**Value**
Column dimensions	360 µm I.D. x 30 mm
Estimated column volume	3.06 µL
Column void volume *(a)*	0.37 ± 0.02 µL
Residence time (at 10 $µL\cdot min^{-1}$)	2.2 ± 0.1 s
IMER Characteristics	
**Description**	**Value**
IMER surface area *(a)*	54 ± 2 cm^2
Coupled protease *(b)*	102 ± 3 µg
Enzyme surface coverage	1.89 ± 0.05 $µg\cdot cm^{-2}$
Specific pepsin loading	31.9 ± 1.3 $µg\cdot mg^{-1}$

(a) Calculated based on 3.2 mg loaded particles with an average particle diameter of 2.86 µm ± 4% CV (coefficient of variance), microsphere density of 1.19 $g\cdot cm^{-3}$ and $1.68\cdot 10^9$ $particles\cdot mL^{-1}$ according to the manufacturer's technical data sheet. For details, see the Supporting Information. *(b)* Determined by UV/Vis spectroscopy, 95% confidence, N = 6.

According to the manufacturer's technical data sheet the average microsphere radius is $r = 1.43\ E - 6\ m$, while the particle count is $1.68\ E + 09\ particles\ /\ mL$

Microparticle surface area calculation:

$$0.125\ mL\ used = 2.1\ E + 08\ particles \quad (S1)$$

$$A = 4 \cdot \pi \cdot r^2 \quad \text{(S2)}$$

$$A = 4 \cdot \pi \cdot (1.43\ E - 6\ m)^2 = 2.57\ E - 11\ m^2\ /particle \quad \text{(S3)}$$

$$A\ tot. = 2.1\ E + 08\ particles \cdot 2.57\ E - 11 m^2/particle = 5.4\ E - 3\ m^2 \quad \text{(S4)}$$

$$A\ tot. = 5.4\ E - 3\ m^2 = 54\ cm^2 \quad \text{(S5)}$$

$$specific\ area = \frac{5.4\ E{-}3\ m^2\ /column}{3.125\ E{-}3\ g\ (particles)} = 1.73\ m^2/g \quad \text{(S6)}$$

6 Peptide Mapping LC-MS, Data Dependent Acquisition and Peptide Identification

The QExactive HESI source was operated at sprayer position C, 320°C capillary temp., + 3.3 kV positive mode and 50 a. u. sheath gas flow. For data dependent acquisition (DDA), MS^1 max. injection time was 100 ms, mass range m/z = 300 to 1500 at 35k resolution (at m/z = 200) and automatic gain control (AGC) target set to $3 \cdot 10^6$. The DDA threshold was $2 \cdot 10^5$ with Top N5, isolation window m/z = 3.0 and dynamic exclusion for 15 seconds. MS^2 max. injection time was 200 ms with 10^6 AGC target at 17.5k resolution and m/z = 120 fixed first mass. HCD fragmentation was performed at 27% normalized collision energy. The acquired data files were converted to mzML format using MSConvert.[2] Peptide annotation was performed with MassSpecStudio,[3] using the settings: OMSSA+ peptide identification algorithm, 10 ppm MS^1 mass tolerance with S/N > 10, MS^2 mass tolerance 20 ppm, non-specific cleavage with 4 to 30 peptide length and +6 max. charge. The min. intensity for MS^1 features was 10^5, the validation p-value was set to > 0.05 and FDR to < 0.05. Peptide hits were checked manually. These peptide search results are depicted and discussed in Supporting Figures S2, S3, S5, S6 and S7.

7 Qualitative Assessment of IMER Performance

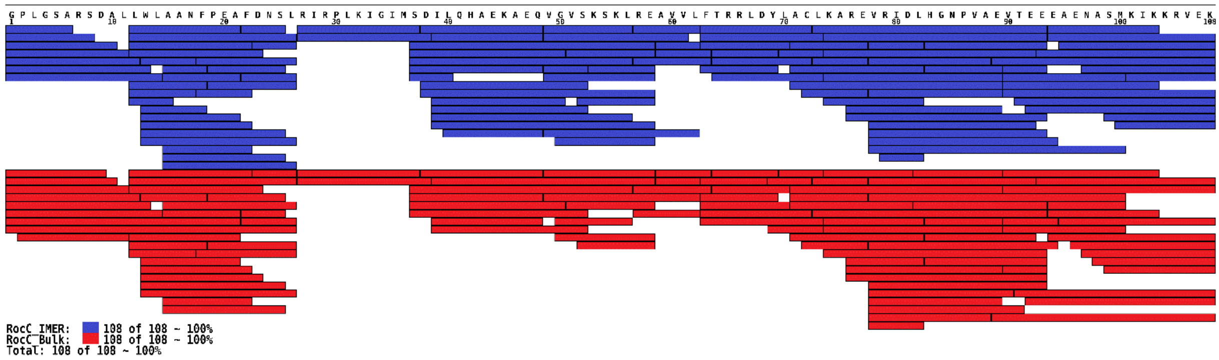

Supporting Figure S2. (Top, blue) peptide map of in-house recombinant RocC (46 µg·mL^{-1} protein in 0.8% FA) digested online with pepsin IMER at 10 µL·min^{-1} and 85 bar back pressure, day 2 of IMER lifetime. (Bottom, red) one hour bulk digestion with 23:1 (w/w) pepsin, LC-MS/MS under identical conditions. Peptide maps were drawn using MS tools.[4]

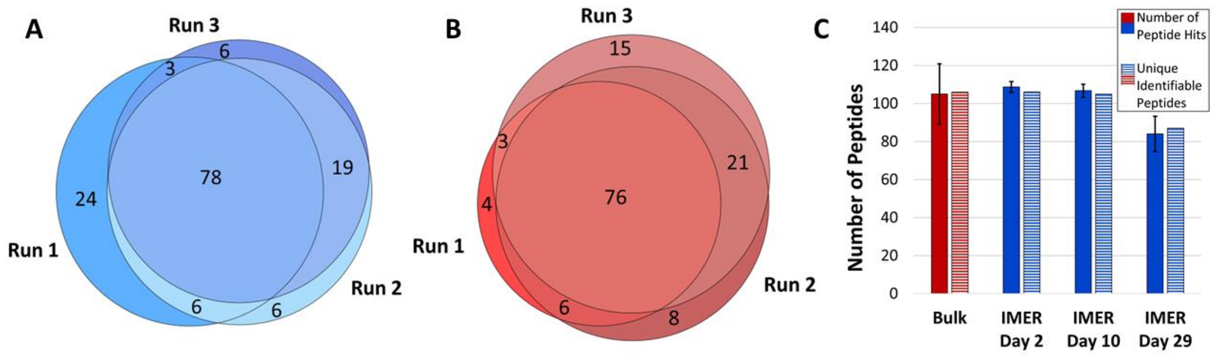

Supporting Figure S3. IMER characterization based on the peptic digestion of in-house recombinant protein RocC: run-to-run reproducibility of peptide hits in three consecutive IMER digestions (Venn diagram A) as well as three replicate peptic digestions in bulk solution (Venn diagram B); total number of peptide hits and number of unique identified peptides detected in bulk digestions and IMER online digestions performed on three different days (C). The error bars represent the confidence interval for 95% confidence, N = 3.

The IMER was utilized to obtain sequence confirmation by peptide mapping of three different in-house recombinantly produced proteins. As the first protein studied, 10 µL samples of the RNA chaperone FinO-domain RocC, at 46 µg·mL^{-1} in 0.8% FA, 1 mM NH_4HCO_3 were digested online, trapped on a 4.0 x 3.0 mm C18 column and desalted (8 min in total). The average pressure during this process was 85 bar. This was followed by data dependent LC-MS/MS analysis in a 10 min gradient (for LC and MS/MS settings see the Experimental Section). The experiments were performed in triplicate consecutively. The LC-MS data were processed and annotated using the freely available Mass Spec Studio software suite.[3] On day 2 of the IMER lifetime, online digestion yielded on average 108.7 ± 2.8 peptide hits per run. Adhering to the criterion that for unambiguous peptide identification each peptide must be present in at least two out of three consecutive runs, a total of 106 peptides were identified. These unique identifiable peptides had an average length of 10.9 amino acids and covered 100% of the 108-residue sequence (comprised of RocC's Fin-O domain and a 5 AAs tag). This excellent digestion efficiency was compared to a standard bulk digest. Here, identical samples (46 µg·mL^{-1} protein

in 0.8% FA) were incubated with additional 2 µg·mL^{-1} pepsin for one hour at room temperature. The digestion was stopped by heat shock at 95°C for 5 min. Three replicate bulk digests were analyzed using the same method and parameters as for the online digestion runs. The bulk digests resulted in an average of 105.0 ± 15.8 peptide hits per run, with 106 unique identifiable peptides of 11.1 amino acids average length. Comparing these results, the IMER required less than three seconds residence time to achieve a comparable hydrolysis level as an one-hour bulk digest at 23:1 protein to protease ratio. Additionally, the inter-run comparability was slightly superior to the bulk digest (Supporting Figures S3A and S3B), given that 78 out of 106 identifiable peptides were present in all three online digestion runs. Both peptide maps derived from the two different digestion methods are compared in Supporting Figure S2.

The above-mentioned online digestion experiment was repeated at IMER lifetime days 10 and 29 to assess the longevity of the surface bound protease. Strikingly, on day 10, the IMER performed almost as well as on day 2 with 106.7 ± 3.5 peptides per run, resulting in 105 unique identifiable peptides with 10.8 average length for RocC. Again, identified peptides span 100% of the protein sequence, demonstrating that no performance decrease had occurred. Only after more than four weeks of storage at 4°C in a fridge, the IMER displayed slightly reduced performance. On IMER lifetime day 29, three consecutive online digestion runs yielded slightly fewer peptide hits: on average 84.0 ± 9.3 per run, of which 86 peptides were unique identifiable. The average peptide length increased marginally to 11.2 residues. However, the IMER was still able to produce 100% sequence coverage for the studied protein. For a graphical comparison of IMER performance over storage time see Supporting Figure S3C.

8 Quantitative Assessment of IMER Performance

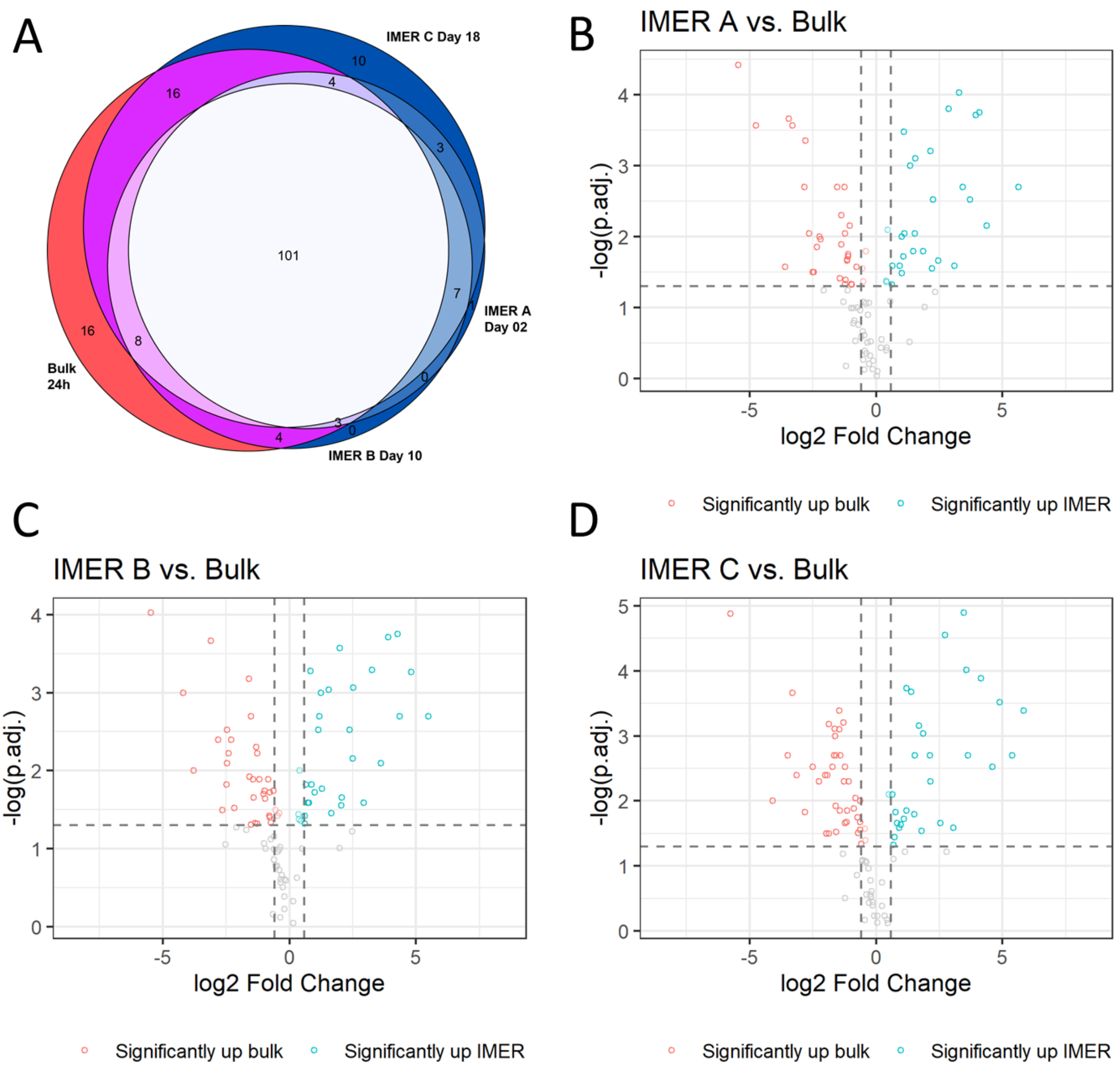

Supporting Figure S4. A: Venn diagram for run-to-run comparison and comparison to 24h bulk digestion for identified peptides. (B-D) Quantitative comparison of peptide amounts between individual IMER experiments and bulk digestion. Significant threshold: adjusted p-value of ≤ 0.05 (-log(p) ≥ 1.3). Peptides showing significant higher amounts in bulk or IMER digestion are colored in red (Bulk) or blue (IMER). Peptides with a fold-change of ≤ 1.5 are represented as red or blue-colored semi-transparent dots and analytes with fold-change of ≥ 1.5 are represented as red or blue-colored dots. N = 3 independent experiments

In absolute numbers, the volcano plots in Supporting Figure S4 reveal significantly more abundant peptides as follows: IMER A vs. bulk: bulk 29 up IMER 27 up, IMER B vs. bulk: bulk 33 up IMER 29 up, and for IMER C vs. bulk: bulk 29 up IMER 30 up.

9 Peptides Maps, Produced by Online IMER Digestion and LC-MS/MS

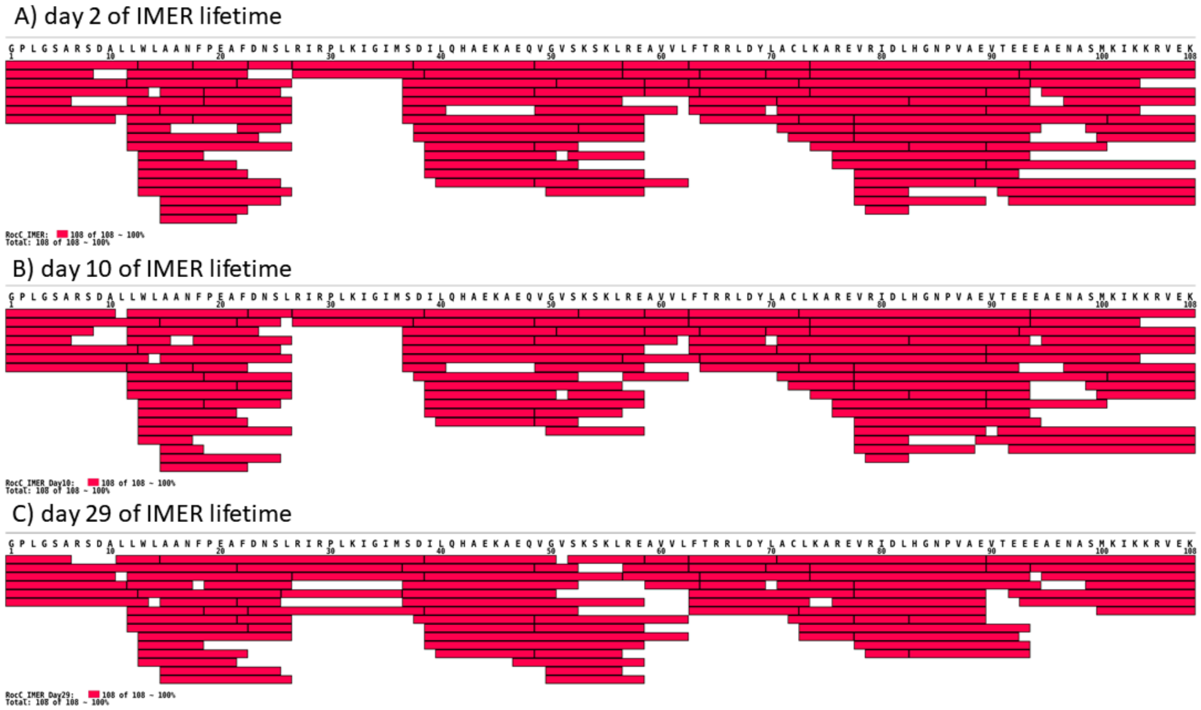

Supporting Figure S5. Peptide maps of in-house recombinant RocC (46 µg·mL^{-1} protein in 0.8% FA) digested online with IMER at 10 µL·min^{-1} and 85 bar, performed at different IMER lifetimes: (A) = IMER lifetime day 2, (B) = day 10, (C) = day 29. For LC-MS/MS settings and information about peptide annotation see the Supporting Information. Each map is created by combining three consecutive runs. Only peptides present in at least two of three runs are considered identified. Peptide maps were drawn using MS Tools.[4]

Peptide Mapping of Recombinant Mal d 1.0201. The produced pepsin IMER was employed for peptide mapping of additional in-house proteins. The 158-residue major apple allergen Mal d 1.0201 was recombinantly produced and analyzed as 50 µg·mL^{-1} samples in 0.8% FA, 2 mM NH_4HCO_3. Two runs were performed at either 5 µL·min^{-1} or 10 µL·min^{-1} respectively. The low flow rate run yielded 110 peptide hits while the run at regular flow rate resulted in 96 peptide hits. 95 of these peptides were found in both runs and therefore, labelled as unique identifiable. The identified peptides had an average length of 8.8 residues and covered 100% of the protein sequence. For peptide map see Supporting figure S6.

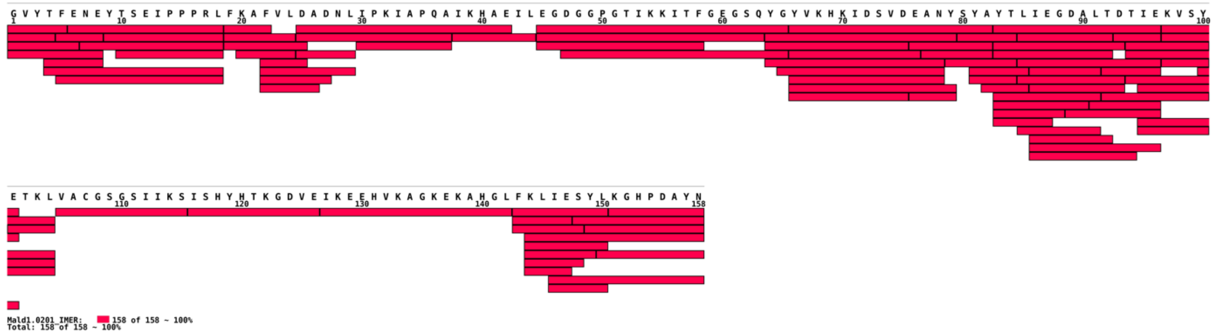

Supporting Figure S6. Peptide map of two combined runs: 10 µL recombinant protein Mal d 1.0201 samples as 50 µg·mL^{-1} solutions in 0.8% FA, 2 mM NH_4HCO_3, digested online using the pepsin IMER. Two runs were performed at either 5 µL·min^{-1} or 10 µL·min^{-1} respectively. Only peptides present in both runs are considered identified. Peptide maps were drawn using MS Tools.[4]

Adding a Chaotropic Agent for Increased Sequence Coverage. 10 µL samples of Act c 8, 122 µg·mL^{-1} in 0.8% FA, 4 mM sodium phosphate were injected in three consecutive runs for online digestion at ambient temperature on day 16 of the IMER lifetime. On average, 73.7 ± 7.5 peptide hits were achieved in each run with 70 unique identifiable peptides of 9.8 average length. However, no full sequence coverage was obtained at an acceptable 85% coverage of the 158-residue protein Act c 8. Given these results, a commonly used chaotropic agent, guanidine hydrochloride (GdnHCl), was employed to assist the protein digestion. Including 3 M GdnHCl in the sample buffer increased the sequence coverage to 100%. At 119.3 ± 9.2 peptide hits per run, more peptides and thus more thorough digestion were observed. 115 of the peptides were present in at least two of three runs and therefore labelled as unique identifiable. The average peptide length was comparable to before with 10.2 amino acids. Furthermore, minor spikes in IMER back pressure were observed upon sample injection of 3 M GdnHCl. Nevertheless, the results were reliable and the microcolumn IMER performed flawlessly. See Supporting Figure S7 for peptide maps.

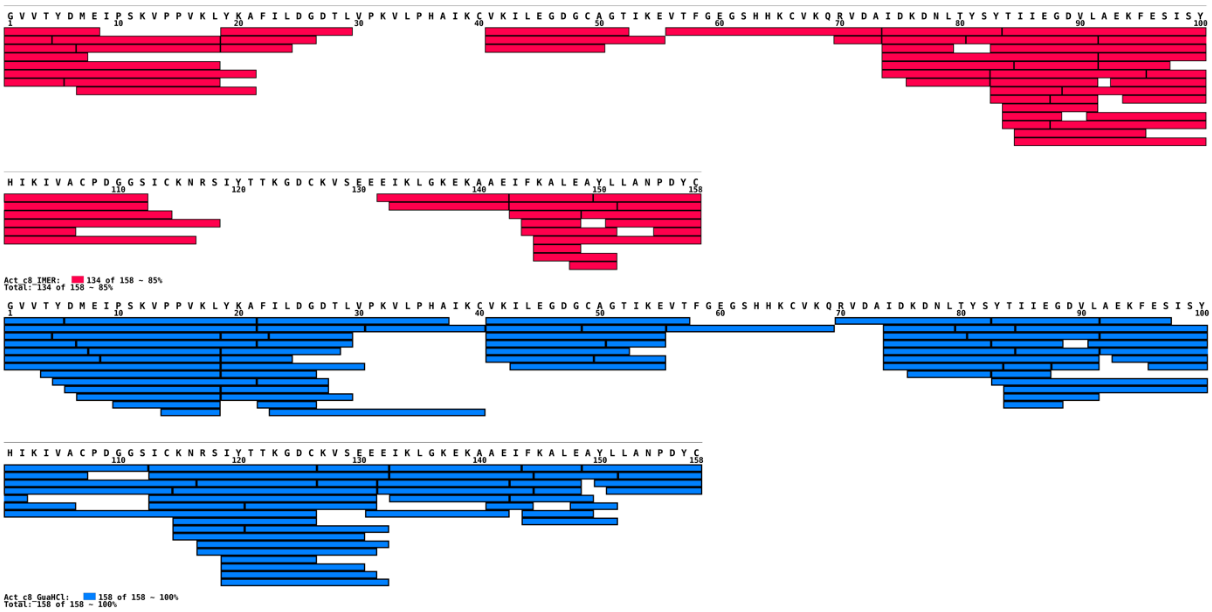

Supporting Figure S7. Peptide maps of recombinant Act c 8 analyzed as 10 µL samples of 125 µg·mL^{-1} protein solution in 4 mM sodium phosphate and 0.8% FA buffer, digested online with the pepsin IMER at 10 µL·min^{-1}. (Top, red), three consecutive runs combined, peptides present in two of the three runs were considered identified. (Blue, bottom) increased sequence coverage after the addition of 3 molar guanidine hydrochloride to the digestion buffer. Peptide maps were drawn using MS Tools.[4]

10 Assessing 3D Printing Reproducibility and Bore Integrity

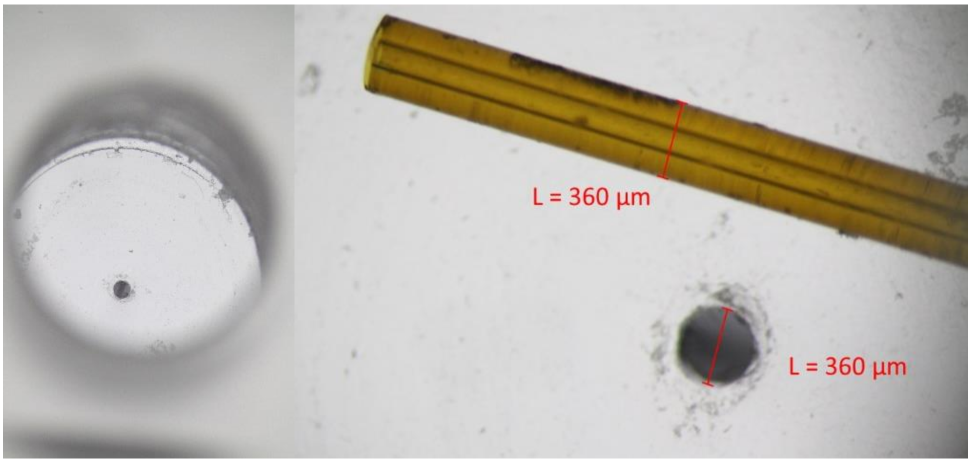

Supporting Figure S8. Microscopic image of the 3D printed microbore column opening on a cured PMMA microcolumn chip (left). size comparison of the microbore on a methanol-washed microcolumn chip with a 360 µm O.D. fused silica capillary (Upchurch Scientific). The inner diameter of the microcolumn was found to be identical to the O.D. of the fused silica capillary, i.e., 360 µm (right).

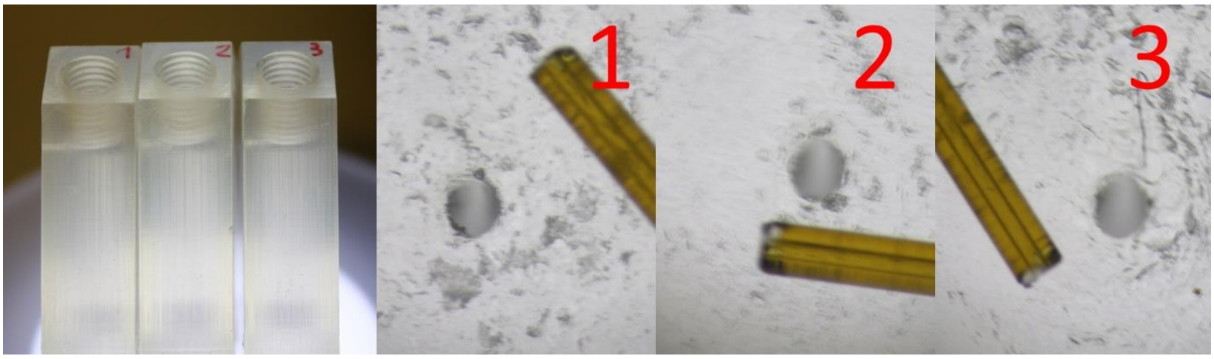

Supporting Figure S9. Comparison of bore diameter for three (left to right) 3D printed microcolumns printed in a single run The inner diameter of the microcolumns was found to be identical to the O.D. of the fused silica capillary, i.e., 360 µm (right).

11 References

(1) Gies, A. P.; Hercules, D. M. Collision Induced Dissociation Study of Ester-Based Polyurethane Fragmentation Reactions. *Anal. Chim. Acta* **2014**, *808*, 199–219.

(2) Chambers, M. C.; Maclean, B.; Burke, R.; Amodei, D.; Ruderman, D. L.; Neumann, S.; Gatto, L.; Fischer, B.; Pratt, B.; Egertson, J.; Hoff, K.; Kessner, D.; Tasman, N.; Shulman, N.; Frewen, B.; Baker, T. A.; Brusniak, M.-Y.; Paulse, C.; Creasy, D.; Flashner, L.; Kani, K.; Moulding, C.; Seymour, S. L.; Nuwaysir, L. M.; Lefebvre, B.; Kuhlmann, F.; Roark, J.; Rainer, P.; Detlev, S.; Hemenway, T.; Huhmer, A.; Langridge, J.; Connolly, B.; Chadick, T.; Holly, K.; Eckels, J.; Deutsch, E. W.; Moritz, R. L.; Katz, J. E.; Agus, D. B.; MacCoss, M.; Tabb, D. L.; Mallick, P. A Cross-Platform Toolkit for Mass Spectrometry and Proteomics. *Nat. Biotechnol.* **2012**, *30* (10), 918–920.

(3) Rey, M.; Sarpe, V.; Burns, K. M.; Buse, J.; Baker, C. A. H.; van Dijk, M.; Wordeman, L.; Bonvin, A. M. J. J.; Schriemer, D. C. Mass Spec Studio for Integrative Structural Biology. *Structure* **2014**, *22* (10), 1538–1548.

(4) Kavan, D.; Man, P. MSTools—Web Based Application for Visualization and Presentation of HXMS Data. *Int. J. Mass Spectrom.* **2011**, *302* (1–3), 53–58.

(6) *Manufacturer technical data sheet (status: November 2021) https://www.polysciences.com/media/amasty/amfile/attach/Juj1wP79rnoBRxZJXrI21bWmlsSJJfiL.pdf
